# Clinician Volume and Outcomes Among Patients Admitted to Nursing Homes for Postacute Care

**DOI:** 10.1001/jamanetworkopen.2025.27234

**Published:** 2025-08-15

**Authors:** Brandi Peacock, Seiyoun Kim, Ziwei Pan, Paul Katz, Hye-Young Jung, Kira Ryskina

**Affiliations:** 1 Division of General Internal Medicine, Department of Medicine, Perelman School of Medicine at the University of Pennsylvania, Philadelphia; 2 Florida State University, College of Medicine, Tallahassee; 3 Division of Health Policy and Economics, Department of Population Health Sciences, Weill Cornell Medical College, New York, New York; 4 Leonard Davis Institute of Health Economics, University of Pennsylvania, Philadelphia

## Abstract

**Importance:**

Little is known about the role of physician and advanced practitioner patient volume in postacute care outcomes in US nursing homes.

**Objective:**

To examine the association between patient volume (the number of nursing home residents seen per year) and patient outcomes.

**Design, Setting, and Participants:**

This cohort study used Medicare Part A and Part B claims and the nursing home minimum data set from 2012 to 2019. Eligible participants were Medicare fee-for-service beneficiaries aged 65 years or older who were admitted to a nursing home for postacute care and seen by a physician (internal medicine, family medicine, general practice, or geriatrics) or advanced practitioner (nurse practitioner or physician assistant) during their stay. The analyses were conducted between October 19, 2022, and June 6, 2025.

**Exposure:**

Treating clinician’s patient panel size.

**Main Outcomes and Measures:**

Patient volume was measured by counting the number of unique patients seen by each clinician and categorized into deciles. Analyses were conducted at the patient-year level. Four risk-adjusted outcomes of care—30-day rehospitalization, emergency department (ED) visit, successful discharge to community, and improvement in functional status at discharge—were measured using Medicare’s quality measures specifications. Poisson regression models with nursing home–level random effects were used to measure the association between each outcome adjusted for patient risk factors and the decile of patient volume. The models also included nursing home characteristics and an indicator for year.

**Results:**

Of the 6 193 638 patient-years in the sample, 3 977 686 (64.2%) were of female and 2 215 952 (35.8%) of male participants; 548 241 patient-years (8.9%) were of Black and 5 376 750 (86.8%) of White participants. Patients of clinicians in the lowest decile of patient volume did not experience significantly different outcomes compared with those in the highest decile: the incidence rate ratio (IRR) was 1.05 (95% CI, 0.76-1.46) for rehospitalizations, 0.96 (95% CI, 0.86-1.07) for successful discharge to community, 1.03 (95% CI, 0.90-1.19) for ED visits, and 0.96 (95% CI, 0.88-1.40) for functional improvement at discharge from the nursing home.

**Conclusions and Relevance:**

In this cohort study of Medicare beneficiaries, we did not find an association between clinician panel size and nursing home postacute care outcomes. These findings may inform the design of care models for clinicians practicing in nursing homes.

## Key Points

**Question:**

Is the patient panel size of a physician or advanced practitioner associated with outcomes of patients admitted to nursing homes for postacute care?

**Findings:**

In this cohort study, nursing home physician or advanced practitioner panel size was not associated with higher rates of rehospitalization, lower rates of successful discharge to community, or lower rates of functional improvement at discharge from postacute care in the nursing home.

**Meaning:**

These results suggest that nursing home physician and advanced practitioner panel size does not play a significant role in postacute care outcomes.

## Teaser

This cohort study of US patients in nursing homes who received postacute care examines the association of nursing home physician or advanced practitioner panel size and rates of rehospitalization, successful discharge to community, and functional improvement at discharge.

## Introduction

Over 1.2 million Medicare beneficiaries receive postacute care in US nursing homes annually.^1^ The outcomes of these patients vary between facilities. For instance, the rate of successful discharge to the community for nursing homes at the 75th percentile of performance is 15 percentage points higher compared with the nursing homes at the 25th percentile.^1^ One possible explanation of the variation may be that some of its key drivers are not well understood. In recent years, calls to concentrate the care of nursing home patients in the hands of nursing home physician specialists have been proposed to translate the enhanced clinical knowledge and increased effectiveness within the nursing home into better patient outcomes.^2,3,4^ However, prior studies on the role of physician specialization in nursing home care quality have not considered patient volume. In this study, we aimed to determine whether nursing home patient volume of a physician or advanced practitioner is associated with outcomes of patients admitted to nursing home for postacute care.

The association of physician volume (ie, the number of unique patients under their care) with outcomes has been documented in several clinical settings. Lower surgeon case volume is associated with worse outcomes for inguinal hernia repair, gastrectomy, abdominal aortic aneurysm repair, and coronary artery bypass, even after accounting for hospital volume and other potentially confounding factors.^5,6,7,8^ Because the typical case volume for surgical procedures varies greatly across conditions and settings, prior studies have evaluated procedure-specific minimum volume thresholds.^9,10,11^ Surgical outcomes require skills and decision making distinct from the care provided by clinicians in nonprocedural specialties.^12^ Studies of the volume-outcomes association for physicians in nonprocedural specialties are less common, and have typically focused on specific conditions or settings.^13,14,15,16^ Those studies may not translate to nursing home clinicians.

Physicians who see patients in nursing homes (ie, nursing home physicians) include generalists (eg, internal medicine and family medicine) and specialists (eg, geriatrics and physical medicine or rehabilitation). Nursing home physicians often see patients in other settings (eg, in the community, hospital).^17^ Moreover, advanced practitioners—nurse practitioners (NPs) and physician assistants (PAs)—provide nearly half of all nursing home visits.^17,18^ Our objectives in this study were to describe the patient panel size (ie, patient volume) for clinicians who practice in nursing homes, and to measure the association between the panel size and the outcomes of patients receiving postacute care in nursing homes.

## Methods

### Data Sources

The Medicare Provider Analysis and Review (MedPAR) file was used to identify patients discharged from a hospital to a nursing home for postacute care between January 1, 2012, and September 30, 2019. MedPAR was used to measure rehospitalizations and patient risk-adjustment variables. These data were linked to information in the carrier file, which contains Medicare Part B claims for physicians and advanced practitioners, to ascertain clinician patient volume and attribute patients to their nursing home clinicians. The outpatient file was used to measure emergency department (ED) visits that did not result in a hospital admission. The nursing home minimum data set (MDS) was used to measure improvement functional status, discharge to the community, and patient characteristics for risk-adjustment. We used the LTCFocus^19^ database and the Medicare Data on Provider Practice and Specialty (MD-PPAS) file to obtain information on nursing home and clinician characteristics.

### Study Population

Our sample included community-dwelling adults ages 65 years or older, who were hospitalized and discharged to a nursing home for postacute care (ie, short-term skilled nursing and/or rehabilitative therapy). We excluded individuals hospitalized from a long-term care nursing home stay and those without continuous Medicare Part A and Part B coverage from hospital admission through 30 days following their postacute care nursing home stay. Because physicians in different specialties take on primary responsibility for medical management of nursing home patients, we included patients under the care of physicians in the following specialties: general practice, family medicine, internal medicine, physical medicine and rehabilitation, geriatrics, and hospital medicine. We also included patients under the care of advanced practitioners (NPs and PAs). Patients without any clinician visits during their nursing home stay were excluded.

### Key Variables

Each patient stay was assigned to the primary clinician in charge of their care based on plurality of all claims during their postacute nursing home stay. Patient volume for each clinician was measured by counting the number of unique patients who were attributed to the clinician during a post-acute care stay in any nursing home. Visit volume was calculated for each year of the study period. Clinician visits to patients outside of nursing homes or visits to patients in long-term care were not considered.

The outcomes and risk-adjustment variables were measured at the patient-year level. For patients with multiple stays in a year, we used the first stay. Improvement in functional status was measured using the 7-item activity of daily living (ADL) scale in MDS, a validated measure of physical function.^20,21,22^ We measured change in functional status as the difference in the ADL score between nursing home admission and discharge assessments.^23^ A change of 1.0 point or more was considered clinically meaningful.^24^

We measured successful discharge to the community, unplanned rehospitalization, and ED visits within 30 days of nursing home admission using the Medicare Quality Measure Technical Specifications.^25^ Successful discharge to community was defined as a discharge from nursing home not followed by a hospitalization, readmission to a nursing home, or death within 30 days. Rehospitalizations and ED visits were measured as mutually exclusive, because most hospitalizations are preceded by an ED visit. ED visits followed by an observation stay were included in the ED visit outcome.

We obtained the following variables measuring nursing home and clinician characteristics, as well as patient demographics and clinical complexity measures used for risk-adjustment. Nursing home characteristics included size, profit status, multifacility chain affiliation, percentage of residents covered by Medicare and by Medicaid, direct-care staffing levels, and urban or rural classification. Clinician characteristics included age, sex, specialty and group size. Patient demographics included age, sex, and race (reported by Medicare beneficiaries and categorized as African American or Black, White, or other race [which included American Indian or Alaska Native, Asian, Hispanic or Latino, Native Hawaiian or other Pacific Islander, and those who identify with 2 or more races]). Clinical complexity variables included the following measures: receipt of Medicare disability benefit, performance on the Cognitive Function Scale,^26^ Elixhauser comorbidity score,^27^ the presence of end-stage kidney disease or dialysis, the number of hospitalizations in prior year, the length of hospital stay, intensive care unit (ICU) days, and 87 additional clinical characteristics from the MDS assessments used by Medicare for risk-adjustment of the outcomes (eTables 1-4 in Supplement 1).^25^

### Missing Data

The variables related to patient, market, and clinician characteristics contained no missing data. Facility characteristics with missing data were filled in using the most recent available value from a prior year (profit status, 6 patient-years [less than 0.01%]; insurance coverage, 29 658 patient-years [0.5%]; and nursing staffing, 1 040 129 patient-years [16.8%]). The observations with any missing data after imputation were omitted from analysis (5678 patient-years [0.1%]).

### Statistical Analysis

Statistical analysis was conducted from October 19, 2022, to June 6, 2025. We first computed the cumulative distribution for the number of patients seen by each clinician. We then categorized clinicians into 2 groups: at or above vs below the median patient volume. We calculated descriptive statistics of the patient, nursing home, and clinician characteristics for the 2 groups. Clinician characteristics were summarized at the clinician-year level. All other analyses were conducted at the patient-year level.

To measure the association of a clinician’s nursing home patient volume with the outcomes, we categorized the sample into deciles based on the distribution of patient volume in the sample. We used Poisson regression with nursing home–level random effects to estimate the incidence rate ratio (IRR) of each outcome for each decile category compared with the highest decile of patient volume. Each model also included an indicator for year, nursing home variables listed above, patient demographics, and a specific subset of the patient-level variables for clinical complexity (eMethods in Supplement 1).

We performed 2 sensitivity analyses: (1) using deciles of case volume determined at the clinician level (given that most nursing home clinicians see only a few nursing home patients per year); and (2) including all patients seen by a clinician to calculate volume (vs only those patients for whom they were the primary clinician). We also performed 2 additional sets of stratified analyses: (1) using volume thresholds calculated separately for urban vs rural settings; and (2) for physicians vs advanced practitioners.

*P* values were from 2-sided tests, and, after accounting for multiple comparisons using the Bonferroni correction, results were deemed statistically significant at the *P* < .003 level. Statistical analyses were conducted with STATA version 18 (StataCorp). The study was approved with a waiver of informed consent by the University of Pennsylvania institutional review board and the Centers for Medicare and Medicaid Services privacy board. We followed the Strengthening the Reporting of Observational Studies in Epidemiology (STROBE) reporting guideline for cohort studies.

## Results

Of the 6 193 638 patient-years in the sample, 3 977 686 (64.2%) were of female and 2 215 952 (35.8%) of male participants; 548 241 patient-years (8.9%) were of Black and 5 376 750 (86.8%) of White participants. There were 77 732 unique clinicians in the sample. The patient-weighted median (IQR) clinician patient volume (ie, the volume of the clinician seen by the median patient in the dataset) was 68 patients per year (31 to 128 patients) (eFigure in Supplement 1). At the clinician level (ie, giving each clinician equal weight regardless of how many patients they saw), the median volume of patients per year was 9 (3-29 patients). Because clinician volume was unevenly distributed (ie, a small proportion of clinicians see many patients, while most see few), the patient-weighted median (68) was higher than the median at the clinician level (9).

Patients treated by lower-volume clinicians were older (aged 85 years or older, 184 445 of 471 776 [39.1%] vs 2 114 482 of 5 721 862 [37.0%]), less likely to have intact cognitive status (257 931 of 471 776 [54.7%] vs 3 476 598 of 5 721 862 [60.8%]), and less likely to have Elixhauser score of 10 or lower (146 236 of 471 776 [31.0%] vs 1 875 508 of 5 721 862 [32.8%]) compared with patients of higher-volume clinicians (Table 1). Lower-volume clinicians were less likely to see patients in larger nursing homes (275 065 of 471 776 [58.3%] vs 3 974 090 of 5 721 862 [69.5%]), more likely to see patients in nonprofit nursing homes (179 220 of 471 776 [38.0%] vs 1 747 425 of 5 721 862 [30.5%]), and less likely to see patients in chain-affiliated nursing homes (256 833 of 471 776 [54.4%] vs 3 337 224 of 5 721 862 [58.3%]). Nursing homes where lower-volume clinicians provided care had a higher percentage of stays covered by Medicaid (median [IQR], 56.4% [35.3%-69.6%] vs 52.2% [30.1%-65.4%]), a lower percentage of Medicare-covered stays (15.4% [9.2%-26.7%] vs 20.4% [12.9%-33.3%]), and were less likely to be located in urban areas (351 932 of 471 776 [74.6%] vs 5 060 513 of 5 721 862 [88.4%]) (Table 1).

**Table 1.** Characteristics of Study Sample

Characteristics	Patient-years, No. (%)		
	Overall (N = 6 193 638)	Lower-volume (n = 471 776)^a^	Higher-volume (n = 5 721 862)^a^
Unique patients, No.^b^	4 784 566	445 750	4 465 173
**Patient characteristics**			
Age, y			
65-74	1 564 524 (25.3)	112 182 (23.8)	1 452 342 (25.4)
75-84	2 330 187 (37.6)	175 149 (37.1)	2 155 038 (37.7)
≥85	2 298 927 (37.1)	184 445 (39.1)	2 114 482 (37.0)
Race			
Black	548 241 (8.9)	39 405 (8.4)	508 836 (8.9)
White	5 376 750 (86.8)	408 463 (86.6)	4 968 287 (86.8)
Other^c^	268 647 (4.3)	23 908 (5.1)	244 739 (4.3)
Sex			
Male	2 215 952 (35.8)	170 376 (36.1)	2 045 576 (35.8)
Female	3 977 686 (64.2)	301 400 (63.9)	3 676 286 (64.2)
Cognitive Function Status (CFS)^d^			
No impairment	3 734 529 (60.3)	257 931 (54.7)	3 476 598 (60.8)
Mild impairment	1 458 248 (23.5)	123 794 (26.2)	1 334 454 (23.3)
Moderate impairment	878 187 (14.2)	78 239 (16.6)	799 948 (14.0)
Severe impairment	122 674 (2.0)	11 812 (2.5)	110 862 (1.9)
Elixhauser Comorbidity Index			
<10	2 021 744 (32.6)	146 236 (31.0)	1 875 508 (32.8)
10 to <20	1 615 882 (26.1)	124 797 (26.5)	1 491 085 (26.1)
20 to <30	1 425 584 (23.0)	111 950 (23.7)	1 313 634 (23.0)
30 to <40	724 089 (11.7)	56 735 (12.0)	667 354 (11.7)
40 or greater	406 339 (6.6)	32 058 (6.8)	37 481 (6.6)
**Nursing home facility characteristics**			
Size			
Small (<50 beds)	449 551 (7.3)	62 711 (13.3)	386 840 (6.8)
Medium (50-99 beds)	1 494 932 (24.1)	134 000 (28.4)	1 360 932 (23.8)
Large (≥100 beds)	4 249 155 (68.6)	275 065 (58.3)	3 974 090 (69.5)
Nonprofit	1 926 645 (31.1)	179 220 (38.0)	1 747 425 (30.5)
Part of a chain	3 594 057 (58.0)	256 833 (54.4)	3 337 224 (58.3)
Urban	5 412 445 (87.4)	351 932 (74.6)	5 060 513 (88.4)
Stays covered by Medicaid, median (IQR), %	52.4 (30.4-65.8)	56.4 (35.3-69.6)	52.2 (30.1-65.4)
Stays covered by Medicare, median (IQR)	20.0 (12.5-33.0)	15.4 (9.2-26.7)	20.4 (12.9-33.3)
Certified nursing assistant (CNA) time per resident, median (IQR), h/d	2.4 (2.1-2.7)	2.4 (2.0-2.7)	2.4 (2.1-2.8)
Registered nurse (RN) time per resident, median (IQR), h/d	0.5 (0.3-0.8)	0.5 (0.3-0.8)	0.5 (0.3-0.8)
Patients admitted for postacute care in a NH per year, median (IQR), No./y	114 (58-204)	58 (24-125)	119 (62-210)

^a^ The median patient volume at the clinician level (9 patients) was used to categorize the sample into 2 groups: lower patient volume (at or below the median) and higher patient volume (above the median).

^b^ The number of unique patients in the low- and high-volume groups does not sum to the overall number of unique patients because some individuals may appear in both groups across different years from 2012 to 2019.

^c^ Other race category includes American Indian or Alaska Native, Asian, Hispanic or Latino, Native Hawaiian or other Pacific Islander, and those who identify with 2 or more races.

^d^ The Cognitive Function Status (CFS) scale is based on the minimum data set 3.0 CFS scale categories.

Fewer lower-volume clinicians were female (60 953 of 147 764 [41.3%] vs of 134 914 [45.5%]), internal medicine (49 720 of 147 764 [33.6%] vs 48 178 of 134 914 [35.7%]), physical medicine and rehabilitation (2596 of 147 764 [1.8%] vs 5068 of 134 914 [3.8%]), geriatric specialists (2431 of 147 764 [1.6%] vs 4613 of 134 914 [3.4%]), or NPs (28 372 of 147 764 [19.2%] vs 38 732 of 134 914 [28.7%]) (Table 2). More lower-volume clinicians specialized in family (50 623 of 147 764 [34.3%] vs 28 197 of 134 914 [20.9%]) or hospital medicine (5466 of 147 764 [3.7%] vs 1802 of 134 914 [1.3%]) (Table 2).

**Table 2.** Nursing Home Clinician Characteristics in the Sample

Characteristics	Overall (N = 282 678)	Clinician-years, No. (%)	
		Lower-volume (n = 147 764)^a^	Higher-volume (n = 134 914)^a^
Unique clinicians, No.^b^	77 732	61 774	38 910
Age, mean (SD), y	51.0 (11.7)	51.5 (12.0)	50.5 (11.4)
Sex			
Male	160 386 (56.7)	86 808 (58.7)	73 578 (54.5)
Female	122 288 (43.3)	60 953 (41.3)	61 335 (45.5)
Specialty or clinician type			
Internal medicine	97 898 (34.6)	49 720 (33.6)	48 178 (35.7)
Family practice	78 820 (27.9)	50 623 (34.3)	28 197 (20.9)
Nurse practitioner	67 104 (23.7)	28 372 (19.2)	38 732 (28.7)
Physician assistant	12 523 (4.4)	5938 (4.0)	6585 (4.9)
Physical medicine and rehabilitation	7664 (2.7)	2596 (1.8)	5068 (3.8)
Hospital medicine	7268 (2.6)	5466 (3.7)	1802 (1.3)
Geriatric medicine	7044 (2.5)	2431 (1.6)	4613 (3.4)
General practice	4357 (1.5)	2618 (1.8)	1739 (1.3)
Practice size			
Solo practice	62 665 (22.2)	32 975 (22.3)	29 690 (22.0)
2-9 clinicians	67 354 (23.8)	34 455 (23.3)	32 899 (24.4)
10-49 clinicians	57 060 (20.2)	28 604 (19.4)	28 456 (21.1)
50-99 clinicians	25 685 (9.1)	13 427 (9.1)	12 258 (9.1)
100-299 clinicians	36 703 (13.0)	19 315 (13.1)	17 388 (12.9)
≥300 clinicians	32 758 (11.6)	18 538 (12.5)	14 220 (10.5)
Unknown^c^	453 (0.2)	450 (0.3)	3 (<0.1)

^a^ The median patient volume at the clinician level (9 patients) was used to categorize the sample into 2 groups: lower patient volume (at or below the median) and higher patient volume (above the median).

^b^ The number of unique clinicians in the low- and high-volume groups does not sum to the overall number of unique clinicians because some clinicians may appear in both groups across different years from 2012 to 2019.

^c^ Four hundred fifty-three clinician-year observations (0.2%) were missing the tax identifier used to calculate group size and their group size was categorized as unknown.

We did not observe a meaningful difference in patient outcomes between the nursing home patient volume categories (Figure). The incidence rate ratio (IRR) for patients of clinicians in the lowest decile (fewer than 13 patients per year) was 1.05 (95% CI, 0.76-1.46; *P* = .76) for rehospitalizations; 0.96 for successful discharge to community (95% CI, 0.86-1.07; *P* = .44), 1.03 (95% CI, 0.90-1.19; *P* = .67) for ED visits; and 0.96 for improvement in functional status (95% CI, 0.88-1.40; *P* = .33), compared with the patients of clinicians in highest decile of patient volume (205 or more patients per year) (Figure).

**Figure.**
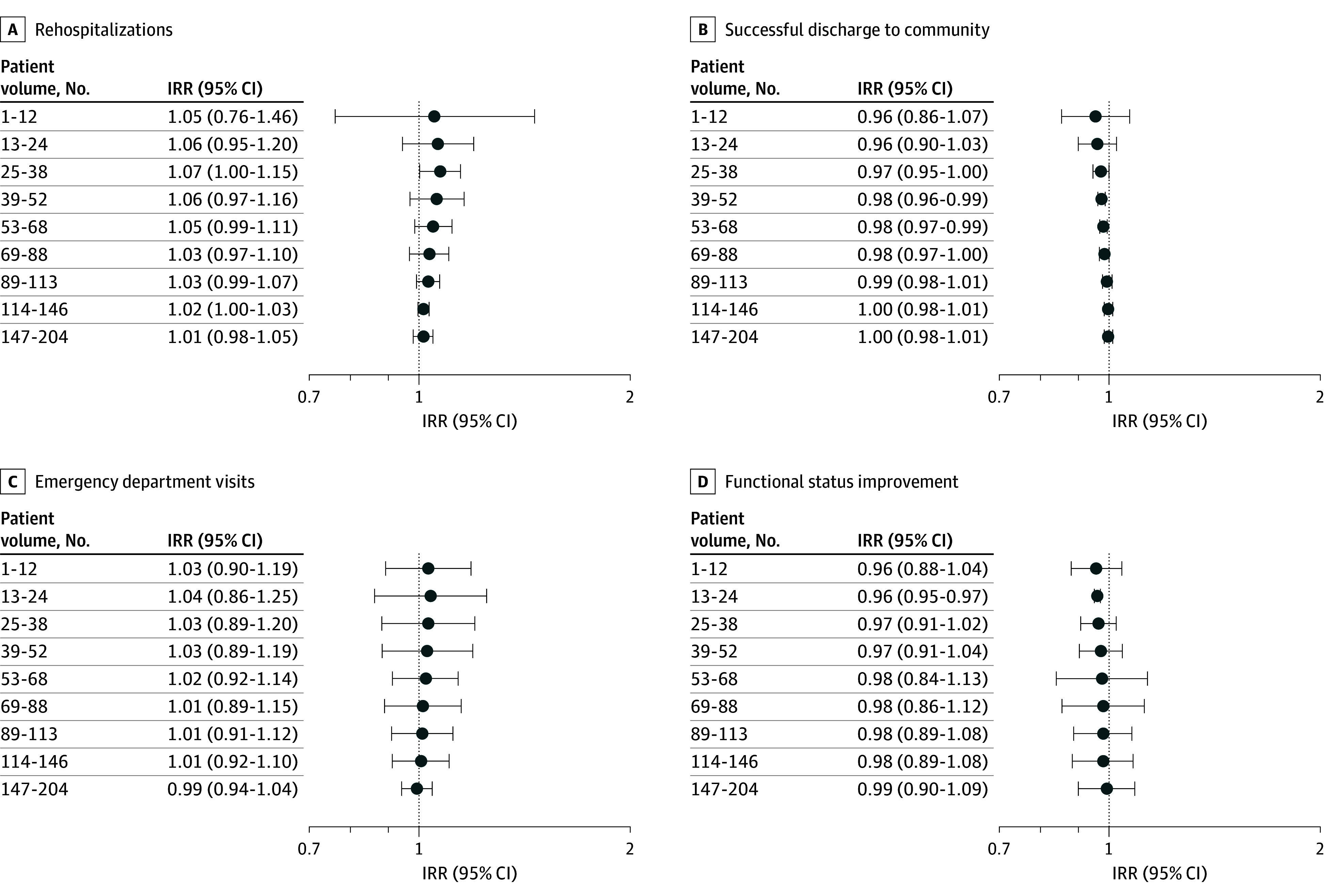
Difference in Rates of Postacute Care Outcomes of Nursing Home Patients by Decile of Patient Volume Compared With Top Decile of Patient Volume The forest plots present the incidence rate ratios (IRRs) for each decile category of patient volume relative to the reference group (top volume category based on the distribution of patients: 205 or more patients), estimated using Poisson regression models with nursing home random effects. Patient volume deciles are based on the distribution of patient volume in the sample. Rehospitalizations and emergency department visits are measured during the 30 days following nursing home admission. Successful discharge to community indicates that the patient was discharged from the nursing home and was not readmitted to the nursing home or hospital or died within 30 days. Functional status improvement indicates improvement in functional status (minimum data set activity of daily living score) of 1 point or more from admission to discharge from the nursing home.

The results of sensitivity analyses using the lower patient volume categories determined by the deciles of patient volume across nursing home clinicians were consistent with the main results (eTable 5 in Supplement 1). When we recalculated patient volume to include any nursing home patient treated by a clinician, we did not observe a pattern between patient volume and outcomes (eTable 6 in Supplement 1).

Consistent with our main findings, we generally did not observe statistically significant differences in the outcomes across patient volume categories for urban and rural nursing homes. One exception was for nursing homes in urban areas: compared with patients of clinicians in the highest decile of patient volume (213 patients per year or more), those treated by clinicians in the lowest decile (less than 15 patients per year) had a lower rate of functional improvement (IRR, 0.95; 95% CI, 0.94-0.97; *P* < .001) (eTable 7 in Supplement 1). The associations between clinician patient volume and outcomes in rural nursing homes were not statistically significant (eTable 8 in Supplement 1).

The results of the subgroup analyses for physicians and advanced practitioners separately were generally consistent with the main findings: we did not find a statistically significant association between outcomes and patient volume (eTable 8 and eTable 9 in Supplement 1). One exception was for successful discharge to community and advanced practitioners: patients in the lowest decile of patient volume had a lower rate of successful discharge to the community (IRR, 0.91; 95% CI, 0.87-0.96; *P* < .001) compared with those treated by advanced practitioners in the highest decile (eTable 9 in Supplement 1).

## Discussion

Physicians and advanced practitioners who treat patients in nursing homes had widely variable patient panel sizes. Postacute care patients of clinicians who saw fewer nursing home patients did not experience meaningfully different outcomes compared with the patients of higher-volume clinicians. Moreover, more than half of nursing home clinicians saw fewer than 10 patients a year. Our findings suggest that efforts to improve outcomes of patients in postacute care in nursing homes are better directed elsewhere. The lack of an association between volume at the clinician level and patient outcomes may be a welcome finding considering that intervention targeting clinician volume such as minimum patient thresholds are not feasible in the nursing home setting.

Prior findings of an association of patient volume with outcomes stemmed primarily from surgical and procedural fields. Adverse outcomes associated with lower case volumes included higher rates of complications,^5,6,28,29^ rehospitalization,^6,29^ reoperation,^6^ recurrence of cancer,^30^ mortality,^8,12^ and higher health care costs.^5^ Volume thresholds and effects were procedure and outcome specific. One notable similarity between our findings is that even relatively low volume requirements would exclude many surgeons performing the procedures, reducing patient access.^9,10^

For nonsurgical specialties, lower-volume clinicians were associated with higher hospital mortality for patients with pulmonary embolism,^14^ lower odds of preventative screening in primary care,^31^ and higher ED visits for patients with diabetes.^32^ In contrast, a study of critical care physicians observed no association between volume and 30-day mortality for patients in intensive care,^33^ and patients of lower-volume physicians hospitalized for heart failure had higher mortality, but lower readmission rates and costs.^13^

In contrast to our study, prior studies of the association of patient volume with outcomes in nursing home settings focused on long-term care residents and evaluated patient volume at the facility rather than individual clinician level. Higher facility volume was associated with a slower decline in residents’ functional status,^34^ and a lower risk of 30-day rehospitalization.^35^ Studies that evaluated characteristics of individual clinicians focused on the proportion of nursing home visits relative to the total visit volume across all settings.^36,37,38^ To our knowledge, our study is the first to report on the association of patient volume with outcomes in nursing homes at the clinician level.

Prior research on patient volume and outcomes includes examples where institutional characteristics matter, others where individual clinician level characteristics matter, and still others that involve a mixture of the 2.^39^ While our analysis did not measure the relative contributions of facility vs individual clinician characteristics, our approach did not identify any time-variant nursing home characteristics that were independently associated with the outcomes, and we did not find that individual clinicians’ patient volume made a difference.

Our findings were insensitive to the approach used to measure patient volume. We observed higher volume to be more consistent with outcomes when only those patients for whom a clinician performed the plurality of visits were considered, although this result was not statistically significant. When we included both patients for whom a clinician was the primary clinician as well as the patients who that clinician saw fewer times than another clinician, there was no association between volume and outcomes. One potential explanation may be that nursing home clinicians who perform cross coverage of patients attributed to other clinicians see more patients in an acute decompensation.

Future research should also evaluate other aspects of physician and advanced practitioner practice in the nursing home such as increased on-site presence of higher-volume clinicians, specific training or expertise, commitment to nursing home practice,^41,42^ or relationships with nursing home direct care staff. These may include the medical staff organizational models employed and the degree of collaboration between advanced practitioners and physicians in the nursing home.

### Limitations

This study had limitations. Nursing home patient volume and other characteristics may confound the association between clinician volume and patient outcomes. Although we controlled for facility size and other nursing home factors known to be associated with patient outcomes, there may have been unobserved patient, clinician, or other nursing home factors that influenced estimates. Moreover, different volumes of patient panels within the same facility may reflect nonrandom patient assignment between clinicians (for example, facility directing more patients to the higher volume clinician). Additionally, our findings may not be generalizable to Medicare Advantage patients, which make up approximately half of Medicare beneficiaries.^40^ Lastly, our study only looks at postacute care patients, future studies should evaluate whether our findings are applicable to long-term care patients.

## Conclusions

We did not observe an association between patient volume and outcomes based on clinician panel size of nursing home postacute care patients. The null results are particularly salient given that many nursing home clinicians have very small patient panels. Efforts to improve postacute care outcomes should consider nursing home characteristics (eg, direct care staffing, processes of care).

## Notes

Supplement 1.
eMethods.
**eFigure.** Cumulative Distribution of the Number of Patients per Clinician **eTable 1.** Complete Regression Estimates: 30-day Rehospitalization **eTable 2.** Complete Regression Coefficients: Successful Discharge to Community **eTable 3.** Complete Regression Coefficients: Emergency Department Visits **eTable 4.** Complete Regression Estimates: Improvement in Functional Status **eTable 5.** Difference in the Rates of Postacute Care Outcomes of NH Patients by Decile of Patient Volume Determined at the Clinician Level **eTable 6.** Difference in the Rates of Postacute Care Outcomes of NH Patients by Decile of Patient Volume Inclusive of All NH Patients Seen by the Clinician **eTable 7.** Differences in the Rates of Postacute Care Outcome Rates Among Nursing Home Patients by Patient Volume Decile: Urban Nursing Homes **eTable 8.** Differences in the Rates of Postacute Care Outcome Rates Among Nursing Home Patients by Patient Volume Decile: Rural Nursing Homes **eTable 9.** Differences in the Rates of Postacute Care Outcome Rates Among Nursing Home Patients by Patient Volume Decile: Nurse Practitioners and Physician Assistants **eTable 10.** Differences in the Rates of Postacute Care Outcome Rates Among Nursing Home Patients by Patient Volume Decile: Physicians

Supplement 2.
Data Sharing Statement
