# Supplementary material for: Clinician Volume and Outcomes Among Patients Admitted to Nursing Homes for Postacute Care
Source: JAMA Netw Open. 2025 Aug 15;8(8):e2527234. doi: 10.1001/jamanetworkopen.2025.27234 (PMC12357193; doi:10.1001/jamanetworkopen.2025.27234)
Supplement: Supplement 1. — eMethods. eFigure. Cumulative Distribution of the Number of Patients per Clinician eTable 1. Complete Regression Estimates: 30-day Rehospitalization eTable 2. Complete Regression Coefficients: Successful Discharge to Community eTable 3. Complete Regression Coefficients: Emergency Department Visits eTable 4. Complete Regression Estimates: Improvement in Functional Status eTable 5. Difference in the Rates of Postacute Care Outcomes of NH Patients by Decile of Patient Volume Determined at the Clinician Level eTable 6. Difference in the Rates of Postacute Care Outcomes of NH Patients by Decile of Patient Volume Inclusive of All NH Patients Seen by the Clinician eTable 7. Differences in the Rates of Postacute Care Outcome Rates Among Nursing Home Patients by Patient Volume Decile: Urban Nursing Homes eTable 8. Differences in the Rates of Postacute Care Outcome Rates Among Nursing Home Patients by Patient Volume Decile: Rural Nursing Homes eTable 9. Differences in the Rates of Postacute Care Outcome Rates Among Nursing Home Patients by Patient Volume Decile: Nurse Practitioners and Physician Assistants eTable 10. Differences in the Rates of Postacute Care Outcome Rates Among Nursing Home Patients by Patient Volume Decile: Physicians [file jamanetwopen-e2527234-s001.pdf]

Supplemental Online Content

Peacock B, Kim S, Pan Z, Katz P, Jung H, Ryskina K. Clinician volume and outcomes among patients admitted to nursing homes for postacute care. *JAMA Netw Open.* 2025;8(8):e2527234. doi:10.1001/jamanetworkopen.2025.27234

eAppendix. Statistical Methods

eFigure. Cumulative Distribution of the Number of Patients per Clinician

eTable 1. Complete Regression Estimates: 30-day Rehospitalization

eTable 2. Complete Regression Coefficients: Successful Discharge to Community

eTable 3. Complete Regression Coefficients: Emergency Department Visits

eTable 4. Complete Regression Estimates: Improvement in Functional Status

eTable 5. Difference in the Rates of Postacute Care Outcomes of NH Patients by Decile of Patient Volume Determined at the Clinician Level

eTable 6. Difference in the Rates of Postacute Care Outcomes of NH Patients by Decile of Patient Volume Inclusive of All NH Patients Seen by the Clinician

eTable 7. Differences in the Rates of Postacute Care Outcome Rates Among Nursing Home Patients by Patient Volume Decile: Urban Nursing Homes

eTable 8. Differences in the Rates of Postacute Care Outcome Rates Among Nursing Home Patients by Patient Volume Decile: Rural Nursing Homes

eTable 9. Differences in the Rates of Postacute Care Outcome Rates Among Nursing Home Patients by Patient Volume Decile: Nurse Practitioners and Physician Assistants

eTable 10. Differences in the Rates of Postacute Care Outcome Rates Among Nursing Home Patients by Patient Volume Decile: Physicians

This supplemental material has been provided by the authors to give readers additional information about their work.

© 2025 Peacock B et al. *JAMA Network Open*.

eAppendix. Statistical Methods

We estimated patient-year level Poisson regression models with NH random effects to examine the association between each of the four outcomes (functional status improvement, 30-day rehospitalization, successful discharge to community, and 30-day ED visit) and clinician patient volume (i.e. panel size). All variables were measured at the patient-year level. For patients with more than one post-acute care stay in a year, we used only the first post-acute care NH stay in the analysis. Standard errors were clustered at the NH level.

We used the following model specification for each of the four outcomes:

$$Y_{i,t} = \beta_1 CaseVolume1_{i,t} + \beta_2 CaseVolume2_{i,t} + \ldots + \beta_9 CaseVolume9_{i,t} + \beta \boldsymbol{X_{i,t}} + \beta \boldsymbol{Z_{i,t}} + a_n + time_t + \boldsymbol{\varepsilon_{i,t}}$$

Where *Y* is the outcome of interest, *CaseVolumeN* is the decile category of patient volume, *X* is a vector of patient characteristics used by CMS for risk adjustment,[1] *Z* is a vector of NH characteristics, a_n is the NH-level random effect, and *time* is a fixed effect indicating year. Each outcome was measured as a dichotomous variable (0 indicating the outcome did not occur, and 1 indicating the outcome occurred).

In the main models, we first assigned each patient-stay to the primary clinician for that stay (the one with the plurality of visits), then calculated the number of unique patients each clinician saw each year. In a sensitivity analysis, we recalculated patient volume for each clinician using all patients seen by each clinician each year. Patient attribution was the same in all analyses: each

© 2025 Peacock B et al. *JAMA Network Open*.

patient-stay was attributed to the clinician with the plurality of visits to that patient during their post-acute care NH stay. We omitted the highest volume decile (10th decile) from the model, which serves as the reference group. Thus, the coefficients $\beta_1, \beta_2, \ldots, \beta_9$ represent the difference in the rate of each outcome for the lower decile categories of patient volume, compared to the highest decile category of patient volume. To facilitate interpretation, we present the incidence rate ratio (IRR).

To implement this, we used the Stata package xtpoisson with the re option.

We conducted four additional sets of analyses using the same estimation approach:

(1) Patient volume categories determined at the clinician level:

Here, we used the distribution of patient volume at the clinician level to define deciles categories using the sample of NH clinicians. Because many clinicians saw a small number of NH patients each year, we conducted this sensitivity analysis to evaluate the association between the categories of volume and patient outcomes for the lowest categories of patient volume. Patients were assigned to the volume category based on the volume of their primary NH clinician. We then estimated the same patient-level model above for the newly calculated lower volume categories, compared to the top patient volume category. Because the thresholds were calculated at the clinician level, the lower volume categories included fewer patients.

(2) Patient volume recalculated to include all patients:

We recalculated patient volume for each clinician in our sample by counting all post-acute care NH patients they saw each year. This included both the patients for whom they were the primary

© 2025 Peacock B et al. *JAMA Network Open*.

clinician as well as the patient they saw fewer times than other clinicians (i.e., cross-coverage patients). After recalculating clinician volume, we attributed each patient to their primary clinician the same way as in the above models (i.e., based on the plurality of claims for that NH stay). We then recategorized the sample into deciles of patient volume and estimated the same patient-level model above for the newly calculated (typically higher) volume categories.

(3) Urban and rural NHs:

This was a stratified analysis of post-acute care outcomes by patient volume conducted within each of the two subgroups separately: (1) patients admitted to NHs in urban settings; and (2) patients admitted to NHs in rural settings. The decile categories of patient volume were calculated within each subgroup separately, because we expected clinicians in rural NHs to have smaller panel sizes. We then estimated the models specified above separately for patients admitted to NHs located in urban areas and for patients admitted to NHs located in rural areas.

(4) Advanced practitioners and physicians:

This was a stratified analysis of post-acute care outcomes by patient volume conducted separately within each of two subgroups: (1) patients attributed to a physician, and (2) patients attributed to an advanced practitioner. We used the same decile volume categories as in our main analysis, because the distribution of patient volume did not differ substantially between the groups. As above, we estimated the same set of models for the four outcomes for patients who were seen by physicians and for patients who were been by advanced practitioners.

© 2025 Peacock B et al. *JAMA Network Open*.

eFigure. Cumulative Distribution of the Number of Patients per Clinician

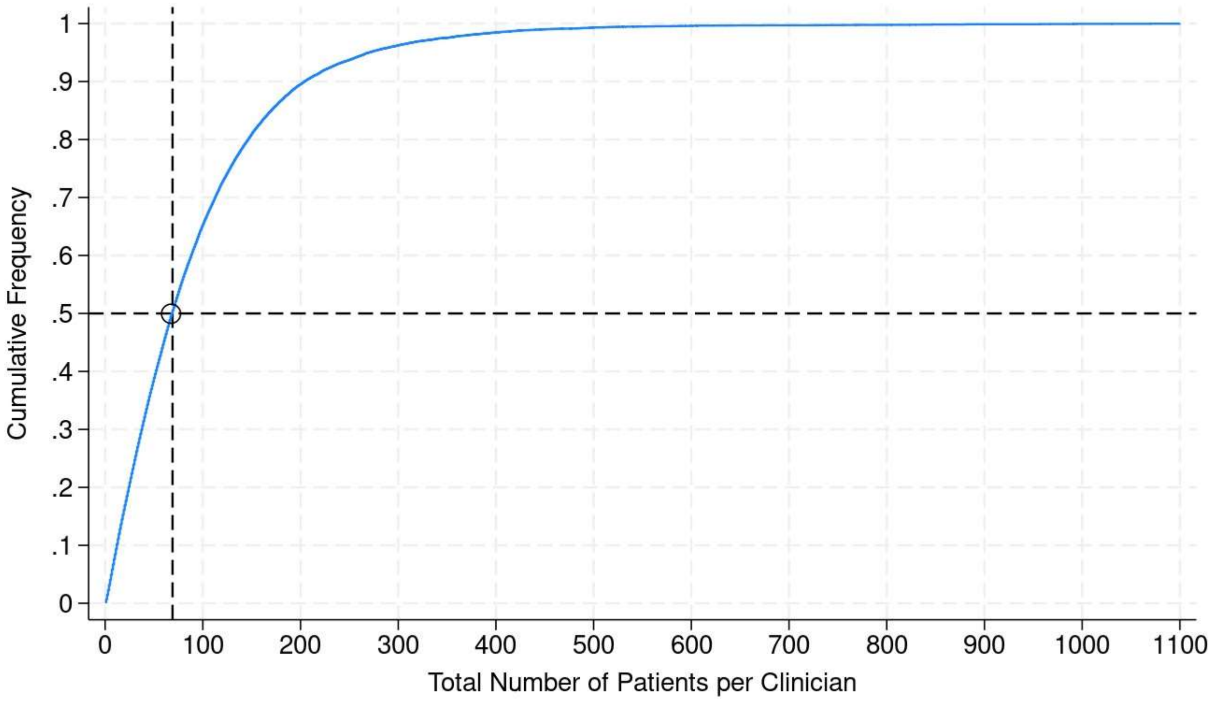

Legend. The x-axis represents the number of patients per clinician. The blue solid line represents the cumulative frequency of patients for clinicians treating an increasing number of patients (i.e., patient-level measure of clinician panel size). The intersection of the dashed line represents the patient-weighted median number of patients per clinician (i.e., the volume of the clinician seen by the median patient in the dataset), which is 68 patients.

© 2025 Peacock B et al. *JAMA Network Open*.

eTable 1. Complete Regression Estimates: 30-day Rehospitalization

	IRR	SE	95% CI	
Volume category (ref. 205+)				
1-12 patients	1.05	0.17	0.76	1.46
13-24 patients	1.06	0.06	0.95	1.20
25-38 patients	1.07	0.03	1.00	1.15
39-52 patients	1.06	0.05	0.97	1.16
53-68 patients	1.05	0.03	0.99	1.11
69-88 patients	1.03	0.03	0.97	1.10
89-113 patients	1.03	0.02	0.99	1.07
114-146 patients	1.02	0.01	1.00	1.03
147-204 patients	1.01	0.02	0.98	1.05
Age	1.00	0.00	1.00	1.00
Year (ref. 2012)				
2013	0.88	0.01	0.86	0.90
2014	0.87	0.01	0.85	0.90
2015	0.87	0.03	0.81	0.93
2016	0.88	0.04	0.82	0.95
2017	0.90	0.05	0.81	1.00
2018	0.90	0.05	0.82	0.99
2019	0.92	0.04	0.85	1.00
Sex: female (ref. male)	0.89	0.01	0.88	0.90
Race: Black (ref. white)	1.05	0.22	0.69	1.60
Race: Other race (ref. white)	1.00	0.11	0.80	1.24
Index hospitalization length of stay	1.01	0.01	0.98	1.03
Days in intensive care unit	1.01	0.02	0.97	1.05
Receipt of Medicare disability benefit	1.03	0.04	0.96	1.11
End-stage renal disease or dialysis	1.29	0.01	1.26	1.32
Number of hospitalizations in prior year	1.10	0.01	1.08	1.13
Elixhauser comorbidity score	1.01	0.00	1.01	1.02
Rarely makes self understood by others (B0700)[a]	1.03	0.08	0.88	1.20
Cognitive status not completely intact (C0100 – C1000)	1.30	0.08	1.12	1.50
Cognitive assessment missing (C0100 and C0600)	1.45	0.09	1.23	1.71
Acute change in mental status (C1600)	1.18	0.13	0.91	1.54
Rejected care for past four to seven days (E0800)	1.13	0.05	1.03	1.25
Wandering once or more in the past week (E0900)	0.99	0.25	0.61	1.61
Walks in room independently or with supervision or limited assistance (E0900 and G0110C)	0.92	0.02	0.89	0.95
Walks in corridor independently or with supervision or limited assistance (E0900 and G0110D)	0.87	0.01	0.86	0.89
Wanders in room or corridor independently or with supervision or limited assistance (E0900 and G0110D)	1.20	0.20	0.82	1.77
Two-person support needed with one or more activities of daily living (G0110A – G0110J)	0.93	0.08	0.80	1.08

© 2025 Peacock B et al. *JAMA Network Open*.

Dependence in eating (G0110H)	1.15	0.03	1.08	1.22
Coughing or choking during meals or when swallowing medications (K0100C)	1.04	0.11	0.83	1.30
Shortness of breath with exertion (J1100A)	1.15	0.10	0.94	1.40
Shortness of breath when sitting at rest (J1100B)	1.19	0.01	1.17	1.21
End-stage prognosis (J1400)	0.89	0.08	0.76	1.04
Internal bleeding (J1550D)	1.39	0.12	1.11	1.75
Venous/Arterial ulcer present (M1030)	1.09	0.01	1.07	1.11
Surgical wound (M1040E)	0.89	0.13	0.69	1.14
Ostomy care (H0100C)	1.29	0.10	1.06	1.58
Parenteral/intravenous feeding (K0500A)	1.46	0.07	1.28	1.66
Feeding tube (K0500B)	1.17	0.27	0.70	1.97
Antibiotic received (N0400F)	1.13	0.00	1.12	1.14
Chemotherapy for cancer (O0100A)	1.11	0.06	0.98	1.26
Radiation for cancer (O0100B)	1.26	0.04	1.16	1.36
Oxygen therapy (O0100C)	1.19	0.07	1.05	1.36
Ventilator or respirator (O0100F)	0.97	0.20	0.65	1.45
Intravenous medications (O0100H)	1.02	0.11	0.82	1.26
Transfusions (O0100I)	1.11	0.05	1.01	1.21
Respiratory therapy (O0400D)	1.08	0.04	0.99	1.18
Cancer (I0100)	0.94	0.01	0.92	0.96
Anemia (I0200)	1.09	0.05	0.99	1.21
Ulcerative Colitis/Crohn’s disease (I1300)	1.01	0.07	0.88	1.16
Viral hepatitis (I2400)	1.05	0.02	1.01	1.09
Alzheimer's disease (I4200)	0.91	0.01	0.90	0.93
Non-Alzheimer's dementia (I4800)	0.95	0.01	0.92	0.97
Seizure disorder or epilepsy (I5400)	0.97	0.02	0.93	1.01
Returned to NH following hospitalization (A1700 and A1800)	1.19	0.15	0.90	1.59
First assessment was for significant change in status (A0310A)	1.09	0.03	1.03	1.15
Facility size (ref. Small)				
Medium	1.02	0.30	0.57	1.82
Large	1.04	0.44	0.44	2.46
For-profit status	1.04	0.09	0.87	1.25
Multi chain facility	0.99	0.10	0.82	1.20
Urban (vs. rural)	1.12	0.13	0.87	1.45
% Medicaid stays	1.00	0.00	1.00	1.00
% Medicare stays	1.00	0.00	1.00	1.00
Registered nurse HPRD[b]	0.98	0.09	0.82	1.18
Licensed practical nurse HPRD	1.03	0.16	0.75	1.40
Certified nursing assistant HPRD	0.99	0.05	0.90	1.09

[a] MDS 3.0 item number in parenthesis
[b] HPRD, hours per resident day

© 2025 Peacock B et al. *JAMA Network Open*.

eTable 2. Complete Regression Coefficients: Successful Discharge to Community

	IRR	SE	95% CI	
Volume category (ref. 205+)				
1-12 patients	0.96	0.06	0.86	1.07
13-24 patients	0.96	0.03	0.90	1.03
25-38 patients	0.97	0.01	0.95	1.00
39-52 patients	0.98	0.01	0.96	0.99
53-68 patients	0.98	0.01	0.97	0.99
69-88 patients	0.98	0.01	0.97	1.00
89-113 patients	0.99	0.01	0.98	1.01
114-146 patients	1.00	0.01	0.98	1.01
147-204 patients	1.00	0.01	0.98	1.01
Age	1.00	0.00	0.99	1.00
Year (ref. 2012)				
2013	1.03	0.01	1.00	1.06
2014	1.03	0.02	0.99	1.08
2015	1.02	0.03	0.97	1.07
2016	1.01	0.02	0.98	1.05
2017	0.99	0.01	0.96	1.01
2018	0.96	0.01	0.95	0.98
2019	0.97	0.01	0.95	0.99
Sex: female (ref. male)	1.01	0.00	1.01	1.01
Race: Black (ref. white)	1.02	0.08	0.88	1.18
Race: Other race (ref. white)	1.04	0.01	1.02	1.07
Index hospitalization length of stay	0.99	0.00	0.98	0.99
Days in intensive care unit	1.00	0.00	1.00	1.00
Receipt of Medicare disability benefit	0.96	0.01	0.94	0.99
End-stage renal disease or dialysis	0.77	0.01	0.76	0.78
Number of hospitalizations in prior year	0.94	0.00	0.94	0.95
Elixhauser comorbidity score	1.00	0.00	1.00	1.00
Rarely makes self-understood by others (B0700)	0.97	0.01	0.95	1.00
Ability to understand others (B0800)	0.95	0.01	0.94	0.97
Vision impairment (B1000)	0.98	0.03	0.92	1.04
Cognitive status not completely intact (C0100 – C1000)	0.92	0.03	0.87	0.98
Acute change in mental status (C1600)	0.91	0.01	0.89	0.93
Cognitive Function Score (MDS 3.0-CFS) (ref. no impairment)				
Mild impairment	0.90	0.02	0.87	0.93
Moderate impairment	0.85	0.02	0.82	0.89
Severe impairment	0.80	0.02	0.77	0.83
MDS_ADL score	0.98	0.00	0.97	0.99
Any potential indicators of psychosis or behavioral symptoms (E0100 and E0200)	0.86	0.02	0.82	0.90

© 2025 Peacock B et al. *JAMA Network Open*.

Wandering once or more in the past week (E0900)	0.90	0.02	0.87	0.93
Dependence in transfer (G0110B)	1.01	0.01	1.00	1.02
Dependence in walking in room (G0110C)	1.05	0.01	1.04	1.07
Dependence in walking in corridor (G0110D)	1.04	0.01	1.01	1.07
Dependence in locomotion on unit (G0110E)	0.98	0.03	0.92	1.03
Dependence in dressing (G0110G)	0.97	0.01	0.95	0.99
Dependence in eating (G0110H)	0.92	0.00	0.92	0.93
Dependence in toilet use (G0110I)	1.00	0.00	0.99	1.01
Dependence in personal hygiene (G0110J)	0.95	0.00	0.94	0.96
Depending in bathing (G0120)	1.00	0.01	0.99	1.01
Balance moving from standing to seated position (G0300A)	1.02	0.01	1.00	1.03
Balance walking (G0300B)	1.03	0.02	1.00	1.07
Any condition related to intellectual/developmental disability status (A1550)	1.06	0.02	1.01	1.11
Urinary incontinence (H0300)	0.97	0.00	0.96	0.97
Shortness of breath with exertion (J1100A)	0.97	0.01	0.94	0.99
Shortness of breath when sitting at rest (J1100B)	0.91	0.00	0.90	0.92
Shortness of breath when lying flat (J1100C)	0.97	0.00	0.96	0.97
Any swallowing disorder (K0100)	0.98	0.00	0.97	0.99
Weight loss (K0300)	0.97	0.00	0.96	0.98
Wound infection (I2500)	0.93	0.00	0.92	0.94
Hemiplegia (I4900)	0.99	0.00	0.98	1.00
Paraplegia (I5000)	0.91	0.02	0.86	0.95
Quadriplegia (I5100)	0.82	0.03	0.77	0.88
Multiple Sclerosis (I5200)	1.00	0.02	0.96	1.04
Huntington’s disease (I5250)	0.99	0.04	0.92	1.06
Seizure disorder or epilepsy (I5400)	1.01	0.00	1.00	1.01
Infection of the foot (M1040A)	0.93	0.00	0.92	0.93
Diabetic foot ulcer (M1040B)	0.87	0.01	0.86	0.88
Surgical wound (M1040E)	1.10	0.01	1.07	1.13
Parenteral/intravenous feeding, feeding tube, or mechanically altered diet (K0500A–C)	0.95	0.01	0.92	0.97
Maximum number of injections (N0300 and N0350A)	1.00	0.00	1.00	1.00
Chemotherapy for cancer (O0100A)	0.93	0.02	0.90	0.97
Radiation for cancer (O0100B)	0.79	0.01	0.78	0.81
Oxygen therapy (O0100C)	0.97	0.00	0.96	0.97
Suctioning (O0100D)	0.84	0.04	0.77	0.91
Tracheostomy (O0100E)	0.90	0.02	0.86	0.94
Ventilator or respirator (O0100F)	1.04	0.01	1.02	1.05
Transfusions (O0100I)	0.98	0.00	0.97	0.98
Dialysis (O0100J)	1.11	0.01	1.09	1.12
Anemia (I0200)	0.98	0.00	0.98	0.99
Heart failure (I0600)	0.96	0.00	0.96	0.97
Hypertension (I0700)	1.01	0.00	1.00	1.01

© 2025 Peacock B et al. *JAMA Network Open*.

Pneumonia (I2000)	1.03	0.01	1.01	1.05
Viral hepatitis (I2400)	0.94	0.01	0.91	0.96
Diabetes mellitus (I2900)	0.99	0.00	0.99	1.00
Hyperkalemia (I3200)	0.94	0.01	0.93	0.96
Hip fracture (I3900)	1.02	0.02	0.98	1.06
Other fracture (I4000)	1.02	0.00	1.01	1.02
Alzheimer's disease (I4200)	0.98	0.02	0.95	1.02
Non-Alzheimer's dementia (I4800)	0.97	0.01	0.95	0.98
Malnutrition (I5600)	0.95	0.04	0.88	1.04
Anxiety disorder (I5700)	0.97	0.00	0.97	0.98
Manic depression (I5900)	0.95	0.02	0.92	0.99
Psychotic disorder (I5950)	0.92	0.07	0.80	1.06
Schizophrenia (I6000)	0.88	0.14	0.67	1.16
Asthma, chronic obstructive pulmonary disease, chronic lung disease (I6200)	0.99	0.00	0.98	1.00
Facility size (ref. Small)				
Medium	1.04	1.60	0.04	24.20
Large	1.04	1.80	0.03	35.21
For-profit status	1.01	0.12	0.80	1.28
Multi chain facility	0.99	0.02	0.95	1.04
Urban (vs. rural)	1.01	0.06	0.89	1.14
% Medicaid stays	1.00	0.00	0.99	1.01
% Medicare stays	1.00	0.01	0.99	1.01
Registered nurse HPRD[b]	1.00	0.26	0.60	1.66
Licensed practical nurse HPRD	0.98	0.11	0.78	1.23
Certified nursing assistant HPRD	1.01	0.06	0.89	1.15

[a] MDS 3.0 item number in parenthesis

[b] HPRD, hours per resident day

© 2025 Peacock B et al. *JAMA Network Open*.

eTable 3. Complete Regression Coefficients: Emergency Department Visits

	IRR	SE	95% CI	
Volume category (ref. 205+)				
1-12 patients	1.03	0.07	0.90	1.19
13-24 patients	1.04	0.09	0.86	1.25
25-38 patients	1.03	0.08	0.89	1.20
39-52 patients	1.03	0.08	0.89	1.19
53-68 patients	1.02	0.05	0.92	1.14
69-88 patients	1.01	0.06	0.89	1.15
89-113 patients	1.01	0.05	0.91	1.12
114-146 patients	1.01	0.05	0.92	1.10
147-204 patients	0.99	0.03	0.94	1.04
Age	1.00	0.00	0.99	1.00
Year (ref. 2012)				
2013	1.36	0.02	1.31	1.42
2014	1.40	0.03	1.31	1.48
2015	1.45	0.04	1.35	1.55
2016	1.49	0.05	1.36	1.63
2017	1.53	0.04	1.41	1.65
2018	1.55	0.04	1.42	1.70
2019	1.58	0.05	1.43	1.74
Sex: female (ref. male)	0.89	0.00	0.88	0.90
Race: Black (ref. white)	1.06	0.19	0.72	1.55
Race: Other race (ref. white)	0.96	0.33	0.50	1.84
Index hospitalization length of stay	1.00	0.00	1.00	1.01
Days in intensive care unit	1.01	0.00	1.00	1.01
Receipt of Medicare disability benefit	1.09	0.03	1.03	1.16
End-stage renal disease or dialysis	1.39	0.02	1.33	1.45
Number of hospitalizations in prior year	1.08	0.02	1.03	1.13
Elixhauser comorbidity score	1.00	0.00	1.00	1.01
Rarely makes self-understood by others (B0700)	0.86	0.08	0.73	1.02
Cognitive status not completely intact (C0100 – C1000)	1.21	0.01	1.19	1.23
Cognitive assessment missing (C0100 and C0600)	1.29	0.07	1.12	1.49
Acute change in mental status (C1600)	1.20	0.06	1.07	1.35
Rejected care for past four to seven days (E0800)	1.26	0.01	1.24	1.28
Wandering once or more in the past week (E0900)	1.39	0.02	1.33	1.46
Walks in room independently or with supervision or limited assistance (E0900 and G0110C)	1.01	0.03	0.94	1.08
Walks in corridor independently or with supervision or limited assistance (E0900 and G0110D)	0.97	0.03	0.91	1.04
Wanders in room or corridor independently or with supervision or limited assistance (E0900 and G0110D)	1.00	0.02	0.96	1.04

© 2025 Peacock B et al. *JAMA Network Open*.

Two-person support needed with one or more activities of daily living (G0110A – G0110J)	1.03	0.19	0.70	1.49
Dependence in eating (G0110H)	0.96	0.13	0.74	1.25
Coughing or choking during meals or when swallowing medications (K0100C)	1.03	0.06	0.92	1.16
Shortness of breath with exertion (J1100A)	1.02	0.07	0.88	1.18
Shortness of breath when sitting at rest (J1100B)	1.12	0.01	1.09	1.15
End-stage prognosis (J1400)	0.95	0.06	0.85	1.07
Internal bleeding (J1550D)	1.62	0.02	1.57	1.67
Venous/Arterial ulcer present (M1030)	0.92	0.01	0.91	0.93
Surgical wound (M1040E)	1.01	0.03	0.95	1.07
Burn care (M1040f)	1.01	0.06	0.91	1.13
Ostomy care (H0100C)	1.15	0.02	1.11	1.19
Parenteral/intravenous feeding (K0500A)	1.32	0.11	1.08	1.63
Feeding tube (K0500B)	1.52	0.01	1.49	1.54
Anticoagulant medication received (n0400e)	0.98	0.02	0.94	1.02
Antibiotic received (N0400F)	1.12	0.00	1.12	1.13
Oxygen therapy (O0100C)	1.03	0.02	0.99	1.07
Tracheostomy (O0100E)	1.15	0.26	0.69	1.91
Ventilator or respirator (O0100F)	0.91	0.09	0.76	1.09
Intravenous medications (O0100H)	1.01	0.03	0.95	1.06
Transfusions (O0100I)	1.11	0.02	1.07	1.16
Speech Language Pathology and Audiology Services (O0400a4)	1.10	0.04	1.02	1.19
Respiratory therapy (O0400d2)	0.96	0.10	0.78	1.17
Cancer (I0100)	1.02	0.02	0.97	1.06
Viral hepatitis (I2400)	1.12	0.05	1.02	1.23
Seizure disorder or epilepsy (I5400)	1.12	0.03	1.05	1.19
Respiratory failure (i6300)	0.92	0.04	0.85	0.99
Returned to NH following hospitalization (A1700 and A1800)	1.04	0.02	1.00	1.08
First assessment was for significant change in status (A0310A)	0.96	0.02	0.92	1.00
Facility size (ref. Small)				
Medium	1.01	0.20	0.68	1.48
Large	0.93	0.25	0.57	1.52
For-profit status	1.03	0.04	0.95	1.12
Multi chain facility	1.02	0.15	0.75	1.38
Urban (vs. rural)	0.74	0.42	0.32	1.70
% Medicaid stays	1.00	0.00	1.00	1.00
% Medicare stays	1.00	0.00	1.00	1.00
Registered nurse HPRD[b]	0.96	0.20	0.66	1.41
Licensed practical nurse HPRD	1.00	0.02	0.97	1.03
Certified nursing assistant HPRD	1.01	0.01	0.99	1.02

[a] MDS 3.0 item number in parenthesis

[b] HPRD, hours per resident day

eTable 4. Complete Regression Estimates: Improvement in Functional Status

	IRR	SE	95% CI	
Volume category (ref. 205+)				
1-12 patients	0.96	0.04	0.88	1.04
13-24 patients	0.96	0.01	0.95	0.97
25-38 patients	0.97	0.03	0.91	1.02
39-52 patients	0.97	0.04	0.91	1.04
53-68 patients	0.98	0.08	0.84	1.13
69-88 patients	0.98	0.07	0.86	1.12
89-113 patients	0.98	0.05	0.89	1.08
114-146 patients	0.98	0.05	0.89	1.08
147-204 patients	0.99	0.05	0.90	1.09
Age	0.99	0.00	0.99	1.00
Year (ref. 2012)				
2013	1.02	0.03	0.96	1.07
2014	1.01	0.04	0.94	1.09
2015	1.00	0.06	0.89	1.12
2016	1.00	0.06	0.90	1.13
2017	1.03	0.06	0.91	1.15
2018	1.03	0.05	0.93	1.15
2019	1.05	0.05	0.96	1.15
Sex: female (ref. male)	1.02	0.00	1.01	1.02
Race: Black (ref. white)	0.95	0.19	0.66	1.36
Race: Other race (ref. white)	0.99	0.03	0.94	1.06
Index hospitalization length of stay	1.00	0.01	0.98	1.01
Days in intensive care unit	1.00	0.00	1.00	1.00
Receipt of Medicare disability benefit	0.97	0.02	0.93	1.01
End-stage renal disease or dialysis	0.93	0.01	0.90	0.96
Number of hospitalizations in prior year	0.98	0.02	0.94	1.02
Elixhauser comorbidity score	1.00	0.00	1.00	1.00
Rarely makes self understood by others (B0700)[a]	0.73	0.01	0.72	0.75
Cognitive status not completely intact (C0100 – C1000)	0.73	0.07	0.65	0.83
Cognitive assessment missing (C0100 and C0600)	0.79	0.00	0.79	0.80
Acute change in mental status (C1600)	0.97	0.11	0.78	1.19
Rejected care for past four to seven days (E0800)	0.91	0.02	0.88	0.95
Wandering once or more in the past week (E0900)	0.96	0.04	0.88	1.05
Walks in room independently or with supervision or limited assistance (E0900 and G0110D)	1.07	0.07	0.93	1.22
Walks in corridor independently or with supervision or limited assistance (E0900 and G0110D)	1.04	0.02	0.99	1.09
Wanders in room or corridor independently or with supervision or limited assistance (E0900 and G0110D)	0.96	0.09	0.80	1.14
Two-person support needed with one or more activities of daily living (G0110A – G0110J)	0.90	0.13	0.69	1.17

© 2025 Peacock B et al. *JAMA Network Open*.

Dependence in eating (G0110H)	1.08	0.20	0.73	1.60
Coughing or choking during meals or when swallowing medications (K0100C)	0.92	0.12	0.72	1.17
Shortness of breath with exertion (J1100A)	1.01	0.05	0.91	1.12
Shortness of breath when sitting at rest (J1100B)	0.95	0.02	0.92	0.98
End-stage prognosis (J1400)	0.91	0.04	0.83	0.99
Internal bleeding (J1550D)	0.97	0.06	0.85	1.09
Venous/Arterial ulcer present (M1030)	0.79	0.04	0.72	0.86
Surgical wound (M1040E)	1.10	0.06	0.97	1.24
Ostomy care (H0100C)	0.96	0.01	0.94	0.99
Parenteral/intravenous feeding (K0500A)	0.94	0.04	0.86	1.02
Feeding tube (K0500B)	0.94	0.10	0.78	1.14
Antibiotic received (N0400F)	0.97	0.00	0.96	0.98
Chemotherapy for cancer (O0100A)	0.95	0.01	0.94	0.97
Radiation for cancer (O0100B)	0.86	0.01	0.84	0.87
Oxygen therapy (O0100C)	0.99	0.03	0.93	1.06
Ventilator or respirator (O0100F)	1.02	0.01	1.01	1.03
Intravenous medications (O0100H)	1.02	0.07	0.89	1.17
Transfusions (O0100I)	1.01	0.05	0.92	1.12
Respiratory therapy (O0400D)	0.98	0.18	0.69	1.40
Cancer (I0100)	1.03	0.03	0.98	1.09
Anemia (I0200)	1.00	0.02	0.95	1.05
Ulcerative Colitis/Crohn’s disease (I1300)	1.07	0.07	0.93	1.23
Viral hepatitis (I2400)	1.03	0.01	1.02	1.05
Alzheimer's disease (I4200)	0.84	0.06	0.75	0.95
Non-Alzheimer's dementia (I4800)	0.86	0.01	0.85	0.87
Seizure disorder or epilepsy (I5400)	0.97	0.02	0.94	1.00
Returned to NH following hospitalization (A1700 and A1800)	0.76	0.12	0.60	0.96
First assessment was for significant change in status (A0310A)	0.88	0.23	0.56	1.37
Facility size (ref. Small)				
Medium	1.01	1.49	0.05	18.96
Large	0.99	1.15	0.10	9.35
For-profit status	0.99	0.38	0.47	2.06
Multi chain facility	1.00	0.03	0.93	1.06
Urban (vs. rural)	0.97	0.45	0.40	2.36
% Medicaid stays	1.00	0.02	0.97	1.03
% Medicare stays	1.00	0.02	0.96	1.04
Registered nurse HPRD[b]	0.98	0.37	0.48	2.03
Licensed practical nurse HPRD	0.99	0.48	0.39	2.54
Certified nursing assistant HPRD	1.01	0.27	0.60	1.70

[a] MDS 3.0 item number in parenthesis
[b] HPRD, hours per resident day

© 2025 Peacock B et al. *JAMA Network Open*.

eTable 5: Difference in the Rates of Postacute Care Outcomes of NH Patients by Decile of Patient Volume Determined at the Clinician Level

Patient volume categories	Rehospitalization IRR (95% CI)	Successful discharge to community IRR (95% CI)	ED visits IRR (95% CI)	Improvement in functional status IRR (95% CI)
1 patient	1.07 [0.76, 1.53]	0.92 [0.78, 1.08]	1.06 [1.03, 1.09]	0.96 [0.84, 1.10]
2 patients	1.03 [0.69, 1.53]	0.95 [0.82, 1.10]	1.05 [1.02, 1.08]	0.97 [0.85, 1.10]
3 patients	1.02 [0.72, 1.46]	0.96 [0.83, 1.11]	1.04 [1.00, 1.07]	0.97 [0.87, 1.08]
4-5 patients	1.02 [0.62, 1.69]	0.96 [0.85, 1.10]	1.03 [1.00, 1.06]	0.98 [0.96, 0.99]
6-8 patients	1.03 [0.79, 1.34]	0.97 [0.87, 1.08]	1.01 [0.94, 1.08]	0.98 [0.91, 1.04]
9-13 patients	1.03 [0.92, 1.16]	0.97 [0.88, 1.06]	1.03 [0.93, 1.13]	0.98 [0.90, 1.05]
14-22 patients	1.05 [0.97, 1.12]	0.97 [0.90, 1.04]	1.04 [0.94, 1.14]	0.98 [0.87, 1.10]
23-37 patients	1.05 [1.02, 1.08]	0.98 [0.94, 1.02]	1.03 [0.96, 1.09]	0.98 [0.93, 1.03]
38-68 patients	1.03 [1.01, 1.06]	0.98 [0.97, 1.00]	1.02 [0.98, 1.06]	0.99 [0.98, 1.00]
69+ patients	Ref.			

IRR, incidence rate ratio; CI, confidence interval

© 2025 Peacock B et al. *JAMA Network Open*.

eTable 6: Difference in the Rates of Postacute Care Outcomes of NH Patients by Decile of Patient Volume Inclusive of All NH Patients Seen by the Clinician

Patient volume categories	Rehospitalization IRR (95% CI)	Successful discharge to community IRR (95% CI)	ED visits IRR (95% CI)	Improvement in functional status IRR (95% CI)
1-26 patients	0.94 [0.88, 1.00]	0.99 [0.87, 1.14]	1.03 [0.93, 1.13]	1.00 [0.93, 1.07]
27-51 patients	0.99 [0.84, 1.16]	0.99 [0.92, 1.06]	1.04 [1.00, 1.07]	0.99 [0.95, 1.04]
52-76 patients	0.99 [0.86, 1.14]	0.99 [0.95, 1.03]	1.04 [1.01, 1.06]	0.99 [0.98, 1.01]
77-102 patients	1.00 [0.83, 1.19]	0.99 [0.98, 1.00]	1.03 [0.99, 1.06]	1.00 [0.91, 1.09]
103-131 patients	0.99 [0.87, 1.12]	0.99 [0.98, 1.01]	1.03 [1.01, 1.06]	0.99 [0.93, 1.07]
132-164 patients	0.99 [0.90, 1.09]	1.00 [0.98, 1.01]	1.02 [0.99, 1.05]	0.99 [0.94, 1.05]
165-205 patients	0.99 [0.86, 1.13]	1.00 [0.99, 1.01]	1.02 [1.00, 1.04]	0.99 [0.92, 1.07]
206-261 patients	0.99 [0.94, 1.05]	1.00 [0.99, 1.02]	1.02 [0.99, 1.04]	1.00 [0.91, 1.09]
262-361 patients	1.00 [0.92, 1.08]	1.00 [0.99, 1.01]	1.02 [0.98, 1.05]	1.00 [0.92, 1.08]
362+ patients	Ref.			

IRR, incidence rate ratio; CI, confidence interval

© 2025 Peacock B et al. *JAMA Network Open*.

eTable 7. Differences in the Rates of Postacute Care Outcome Rates Among Nursing Home Patients by Patient Volume Decile: Urban Nursing Homes

Patient volume categories	Rehospitalization IRR (95% CI)	Successful discharge to community IRR (95% CI)	ED visits IRR (95% CI)	Improvement in functional status IRR (95% CI)
1-14 patients	1.08 [0.89, 1.31]	0.96 [0.84, 1.09]	1.03 [0.81, 1.30]	**0.95 [0.94, 0.97]**
15-27 patients	1.08 [0.99, 1.17]	0.96 [0.91, 1.02]	1.04 [0.81, 1.34]	0.96 [0.93, 1.00]
28-41 patients	1.07 [1.01, 1.14]	0.98 [0.95, 1.00]	1.03 [0.84, 1.27]	0.97 [0.86, 1.09]
42-56 patients	1.06 [0.98, 1.15]	0.98 [0.97, 0.99]	1.03 [0.87, 1.21]	0.97 [0.85, 1.11]
57-73 patients	1.05 [0.95, 1.16]	0.98 [0.97, 1.00]	1.02 [0.89, 1.17]	0.98 [0.80, 1.20]
74-93 patients	1.03 [0.94, 1.12]	0.99 [0.97, 1.01]	1.01 [0.88, 1.17]	0.98 [0.85, 1.13]
94-118 patients	1.03 [0.95, 1.11]	1.00 [0.98, 1.01]	1.01 [0.89, 1.14]	0.98 [0.85, 1.13]
119-152 patients	1.01 [0.96, 1.06]	1.00 [0.98, 1.02]	1.00 [0.90, 1.10]	0.98 [0.84, 1.15]
153-212 patients	1.01 [0.99, 1.03]	1.00 [0.99, 1.02]	0.99 [0.95, 1.04]	1.00 [0.87, 1.13]
213+ patients	Ref.			

IRR, incidence rate ratio; CI, confidence interval

© 2025 Peacock B et al. *JAMA Network Open*.

eTable 8. Differences in the Rates of Postacute Care Outcome Rates Among Nursing Home Patients by Patient Volume Decile: Rural Nursing Homes

Patient volume categories	Rehospitalization IRR (95% CI)	Successful discharge to community IRR (95% CI)	ED visits IRR (95% CI)	Improvement in functional status IRR (95% CI)
1-6 patients	0.88 [0.25, 3.13]	0.96 [0.88, 1.03]	1.03 [0.67, 1.59]	1.01 [0.95, 1.07]
7-13 patients	0.92 [0.38, 2.26]	0.96 [0.90, 1.03]	1.01 [0.76, 1.33]	1.00 [0.83, 1.21]
14-20 patients	0.96 [0.57, 1.61]	0.96 [0.90, 1.04]	1.02 [0.82, 1.26]	1.00 [0.77, 1.28]
21-30 patients	0.98 [0.62, 1.56]	0.97 [0.94, 1.01]	1.01 [0.77, 1.31]	1.00 [0.82, 1.22]
31-41 patients	1.00 [0.65, 1.53]	0.96 [0.93, 1.00]	1.01 [0.83, 1.23]	0.99 [0.81, 1.22]
42-56 patients	0.97 [0.63, 1.51]	0.97 [0.92, 1.02]	1.01 [0.98, 1.05]	1.01 [0.92, 1.10]
57-75 patients	1.01 [0.71, 1.43]	0.98 [0.89, 1.07]	1.02 [0.98, 1.07]	1.00 [0.96, 1.04]
76-102 patients	1.00 [0.82, 1.21]	0.98 [0.87, 1.10]	1.01 [0.94, 1.09]	1.00 [0.94, 1.05]
103-147 patients	1.00 [0.83, 1.19]	0.99 [0.93, 1.06]	1.01 [0.91, 1.13]	1.00 [0.87, 1.14]
148+ patients	Ref.			

IRR, incidence rate ratio; CI, confidence interval

© 2025 Peacock B et al. *JAMA Network Open*.

eTable 9. Differences in the Rates of Postacute Care Outcome Rates Among Nursing Home Patients by Patient Volume Decile: Nurse Practitioners and Physician Assistants

Patient volume categories	Rehospitalization IRR (95% CI)	Successful discharge to community IRR (95% CI)	ED visits IRR (95% CI)	Improvement in functional status IRR (95% CI)
1-12 patients	1.09 [0.67, 1.75]	**0.91 [0.87, 0.96]**	1.05 [0.99, 1.13]	0.96 [0.88, 1.05]
13-24 patients	1.08 [0.62, 1.89]	0.95 [0.89, 1.01]	1.04 [1.01, 1.08]	0.97 [0.92, 1.03]
25-38 patients	1.09 [0.60, 1.97]	0.96 [0.89, 1.05]	1.04 [1.00, 1.09]	0.98 [0.94, 1.02]
39-52 patients	1.07 [0.65, 1.77]	0.96 [0.87, 1.07]	1.04 [1.01, 1.07]	0.99 [0.93, 1.04]
53-68 patients	1.06 [0.65, 1.74]	0.98 [0.89, 1.08]	1.03 [0.96, 1.12]	0.99 [0.97, 1.02]
69-88 patients	1.03 [0.73, 1.47]	0.98 [0.88, 1.09]	1.02 [0.96, 1.08]	1.00 [0.97, 1.02]
89-113 patients	1.04 [0.67, 1.62]	0.99 [0.91, 1.08]	1.00 [0.92, 1.10]	1.00 [0.97, 1.02]
114-146 patients	1.02 [0.74, 1.40]	1.00 [0.94, 1.06]	1.01 [0.94, 1.09]	0.99 [0.97, 1.01]
147-204 patients	1.02 [0.66, 1.59]	1.00 [0.95, 1.05]	1.01 [0.96, 1.06]	1.01 [0.99, 1.02]
205+ patients	Ref.			

IRR, incidence rate ratio; CI, confidence interval

© 2025 Peacock B et al. *JAMA Network Open*.

eTable 10. Differences in the Rates of Postacute Care Outcome Rates Among Nursing Home Patients by Patient Volume Decile: Physicians

Patient volume categories	Rehospitalization IRR (95% CI)	Successful discharge to community IRR (95% CI)	ED visits IRR (95% CI)	Improvement in functional status IRR (95% CI)
1-12 patients	1.03 [0.78, 1.37]	0.97 [0.8, 1.12]	1.02 [0.84, 1.24]	0.96 [0.80, 1.15]
13-24 patients	1.05 [0.98, 1.12]	0.97 [0.88, 1.07]	1.03 [0.79, 1.34]	0.96 [0.92, 1.00]
25-38 patients	1.06 [1.01, 1.12]	0.98 [0.93, 1.04]	1.02 [0.80, 1.30]	0.96 [0.85, 1.08]
39-52 patients	1.05 [0.99, 1.12]	0.98 [0.95, 1.02]	1.02 [0.81, 1.27]	0.97 [0.81, 1.16]
53-68 patients	1.04 [1.01, 1.07]	0.99 [0.96, 1.01]	1.01 [0.84, 1.20]	0.97 [0.74, 1.27]
69-88 patients	1.04 [1.01, 1.06]	0.99 [0.97, 1.01]	1.00 [0.84, 1.20]	0.97 [0.82, 1.16]
89-113 patients	1.03 [0.99, 1.06]	1.00 [0.98, 1.02]	1.01 [0.88, 1.16]	0.97 [0.82, 1.16]
114-146 patients	1.02 [1.00, 1.04]	1.00 [0.98, 1.01]	1.00 [0.86, 1.17]	0.98 [0.82, 1.17]
147-204 patients	1.01 [0.97, 1.05]	1.00 [0.98, 1.01]	0.99 [0.91, 1.06]	0.99 [0.84, 1.16]
205+ patients	Ref.			

IRR, incidence rate ratio; CI, confidence interval

© 2025 Peacock B et al. *JAMA Network Open*.
