# Supplementary material for: Clinician Volume and Outcomes Among Patients Admitted to Nursing Homes for Postacute Care
Source: JAMA Netw Open. 2025 Aug 15;8(8):e2527234. doi: 10.1001/jamanetworkopen.2025.27234 (PMC12357193; doi:10.1001/jamanetworkopen.2025.27234)
Supplement: Supplement 2. — Data Sharing Statement [file jamanetwopen-e2527234-s002.pdf]

Data Sharing Statement

Data

Data available: No

Additional Information

Explanation for why data not available: Access to the Medicare claims and Minimum Data Set (MDS) data used in this study is restricted and subject to the regulations and policies of the Centers for Medicare and Medicare Services. Therefore, the data cannot be shared publicly without explicit permission from CMS. For further inquiries, please contact the corresponding author.
